# Can scientists and knowledge keepers sit comfortably together? An Indigenous physician’s reflections on a decade of participatory research into First Nations nutrition, environment and health

**DOI:** 10.17269/s41997-021-00543-2

**Published:** 2021-06-28

**Authors:** Evan Adams

**Affiliations:** First Nations and Inuit Health Branch, Department of Indigenous Services Canada, Ottawa, Ontario Canada

**Keywords:** Indigenous health, Collaborative participatory research, Ethical space, Respect, Shared knowledge, Modern diet, Environment, Santé autochtone, recherche participative collaborative, espace éthique, respect, connaissances partagées, alimentation moderne, environnement

## Abstract

The author, an Indigenous physician, offers his reflections on the history of scientific research with Indigenous People and its past role in ethical breaches and excesses of colonialism, as a backdrop to the relatively recent advances in collaborative, community-based participatory research involving First Nations and Inuit in Canada. The First Nations Food, Nutrition and Environment Study (2008–2018), introduced in this Special Issue, is presented as an example of an *ethical space* that was sustained for a decade to collaboratively develop new knowledge by First Nations and scientists working together, respectfully and inspired by shared interest. A short overview of twelve articles of the Special Issue is provided and characterized as creating a previously inaccessible picture of the modern diets of First Nations, along with the suite of environmental factors that are present in food and water in and around communities. Ultimately, the author hopes that Canadian society can set the table with Indigenous Peoples and respectfully set opinions onto each other and do this over and over again. With Canada already being a multicultural and pluralistic society, adding Indigenous realities into the mix only respects and honours the Indigenous roots of this country.

## Résumé

L’auteur, médecin autochtone, nous livre ses réflexions sur l’histoire de la recherche scientifique avec les peuples autochtones et le rôle que celle-ci a pu avoir dans les violations éthiques et les excès du colonialisme, comme toile de fond aux avancées relativement récentes de la recherche participative collaborative et communautaire avec les Premières Nations et les Inuits au Canada. L’Étude sur l’alimentation, la nutrition et l’environnement des Premières Nations (2008–2018) qui est présentée dans ce numéro spécial, est citée comme un exemple d’un espace éthique maintenu pendant une décennie pour développer de nouvelles connaissances, de manière collaborative, conjointement par les Premières Nations et les scientifiques qui, mus par un intérêt commun, travaillent ensemble dans le respect. L’auteur nous fournit un bref aperçu des douze articles du numéro spécial qui est considéré comme dressant un tableau, jusqu’alors inexistant, des régimes alimentaires modernes des Premières Nations, ainsi que de la suite des facteurs environnementaux présents dans les aliments et l’eau dans et autour des communautés. L’auteur espère que la société canadienne pourra s’asseoir à la table avec les peuples autochtones et faire se confronter, avec respect, les opinions des uns et des autres, et ce, à répétition. Le Canada étant déjà une société multiculturelle et pluraliste, l’ajout des réalités autochtones ne fait que respecter et honorer les racines autochtones de ce pays.

It is my pleasure to introduce the special issue of the *Canadian Journal of Public Health* that is wholly dedicated to the results of the First Nations Food, Nutrition and Environment Study (FNFNES). FNFNES is the first total diet study conducted with and for First Nations. The study has been designed and realized in partnership with First Nations people, who were participants, investigators, and users of the research results.

This unique 10-year initiative has benefited from ideas of the ecosystem approach to health research, with principles of transdisciplinarity, participation, and equity (Lebel, 2003) embedded within the project. FNFNES brought together First Nations, academia, government, and private laboratories in a joint effort to investigate food quality, food security, exposure to environmental contaminants, and presence of contaminants in water in a statistically representative sample of First Nations people in 92 communities across all regions of Canada south of the 60^th^ parallel.

Over the years and into the twenty-first century, Indigenous voices around the globe have expressed their mistrust of academic researchers, who sometimes would come to communities, impose their agendas into Knowledge Keepers’ time, mine them for information and data, raise expectations, and then disappear—never to be seen again.

At its worst, ethical breaches and medical atrocities were sometimes committed, and deep distrust was cultivated. For instance, Ian Mosby, a food historian and postdoctoral fellow at the University of Guelph, revealed details of highly unethical nutrition experiments (*mal*nutrition experiments, really) performed on Canadian Aboriginal children at six residential schools between 1942 and 1952 (MacDonald et al., 2014).

Linda Tuhiwai Smith introduced her inspirational work “Decolonizing Methodologies: Research and Indigenous Peoples” (1999) with the following reflection: *The ways in which scientific research is implicated in the worst excesses of colonialism remains a powerful remembered history for many of the world’s colonized peoples.*

And this very evident distrust of the academic research enterprise was still quite palpable among First Nations communities in the first decade of this century.

It is not uncommon for Indigenous peoples to be stewards of their ancestral territories and to lament the degradation and contamination of their lands and waters. Often they will articulate how their relationships with their territories have changed as a result. Are country foods even bad for us now? They’ve changed so much.

Despite this apprehension about research and changing lands and waters, some research initiatives in the late 1990s have demonstrated the exciting prospects of academic and Indigenous researchers (with intertwining identities at times) working together, either supported by the Northern Contaminants Program (https://www.ic.gc.ca/eic/site/063.nsf/eng/h_7A463DBA.html) or conducted in the context of the then newly established First Nations Environmental Contaminants Program (https://www.sac-isc.gc.ca/eng/1583779185601/1583779243216#a1), financially supporting not only the engagement of Indigenous people in research, but Indigenous peoples’ participation—and, at times, leadership—in the design, investigation, and analysis of results and the administration of research. However, most of these projects were relatively short-term.

“How can First Nations and academia work together to develop new knowledge that would incorporate both Indigenous ways of knowing and scientific methodology” was a key question that often permeated discussion among some Canadian environmental health scientists and policy analysts working in domains of academia, government, and Indigenous organizations.

In this context, the ideas about an *ethical space*^1^, which may be created through an honest and sincere engagement of contrasting perspectives of the worldview (Ermine, 2007), were very influential in pointing the clear way to engage in research initiatives combining Indigenous leadership and guidance and science methodologies on a long-term basis (Personal communication, Tikhonov C, 2021). These ideas asserted that the creation of such *ethical space* bridging differing philosophies and approaches would advance a dialogue to set the conditions for an agreement to collaborate on a research initiative modelled on appropriate ethical guidelines and guiding principles.

The participatory design and the presentation in this Special Issue of the results of a decade-long history of the FNFNES may offer a good example of an enduring research partnership between First Nations and academia, which in itself represented an *ethical space* of continued dialogues, adjustments, corrections, and improvements in mutual understanding and genuine partnership.

In the first of the 12 articles in this issue, Chan et al. (2021a) describe the fundamental importance of the FNFNES having been designed with respect for the First Nations principles of Ownership, Control, Access, and Possession (OCAP®) (https://fnigc.ca/ocap-training/). The study requested the consideration and resolution of First Nations Chiefs in Assembly in 2007 and was guided by a formal commitment to a long-term partnership on the foundation of clear principles. Chan and colleagues further proceed to describe in considerable detail the rationale, the participatory nature of the methodology, and the lessons learned during the FNFNES study as it was implemented in eight Assembly of First Nation regions, which included the entirety of Canada south of the 60^th^ parallel.

The second article, by Batal et al. (2021a), describes traditional food (TF) systems of First Nations in Canada, including food choices, as well as barriers and supportive factors to healthy diets. These components of the study used a combination of 24-hour recall and food frequency questionnaires to learn about portion sizes and establish the frequency of TF intake in order to allow comparisons across regions. An analysis and comparison by ecozones is also included in the article and is a rather innovative way of assessing traditional food intake in the context of ecozones, which are understood as areas *where organisms and their physical environment endure as a system* (Wiken, 1986).

In the subsequent three manuscripts, Batal and colleagues expand analysis of First Nations diets along several dimensions by: assessing the diet quality using percentage energy from TF and ultra-processed products (UPP) (Batal et al., 2021b); examining whether First Nations adults are meeting their nutrient requirements and the contribution of TF to better nutrient intake (Batal et al., 2021c); and, finally, presenting the first comprehensive picture of the prevalence of food insecurity in First Nations households across Canada, while identifying barriers and enablers to TF consumption (Batal et al., 2021d).

To further accentuate the potential of the new dataset developed by FNFNES, Marushka et al. (2021a) investigate the relationships between fish/seafood consumption patterns and food security status among First Nations communities in Canada. They answer a question about the contribution of fish/seafood to daily nutrient requirements and examine barriers to TF access.

Chan et al. (2021b) present results of an analysis of a total of 2061 food samples (different parts and organs) from 221 species that were collected in FNFNES to investigate a large suite of environmental chemicals. This analysis aims to present the best available evidence and estimate whether there is any health risk from these pollutants at the assessed levels of dietary exposure.

Tikhonov et al. (2021) describe the human biomonitoring survey of 3404 First Nations adults who participated in all components of FNFNES and provided hair samples to be analyzed for mercury. Results of the current body burden of mercury are further compared with historical data and current guidelines.

Schwartz et al. (2021a) discuss the results of the drinking water survey for metals of health concern among 1516 First Nations households that participated in this FNFNES component. The study also assessed the levels of metals that have operational guidance and aesthetic objectives in drinking water of First Nations south of the 60^th^ parallel.

In the absence of much information on the levels of pharmaceuticals as emerging environmental contaminants in proximity to First Nations communities, the study by Schwartz et al. (2021b) offers a unique insight into this issue by reporting on the measurements of 43 pharmaceuticals in 95 First Nations across Canada and discussing some of the associated environmental and human health risks.

Batal et al. (2021e) focus on the associations between the self-reported socio-economic and anthropometric factors and self-reported health status. The study discusses the prevalence of diabetes and obesity and their associations in participants from the FNFNES.

Marushka et al. (2021b) investigate the relationship between dietary exposure to selected persistent organic pollutants (POPs) from traditionally harvested fish consumption and the prevalence of type 2 diabetes in First Nations adults. While this study confirms previous findings that dietary POP exposure may increase the risk of type 2 diabetes, the balance of risk and benefit associated with fish consumption is driven by geographical differences in POP concentrations in fish.

In their totality, the 12 articles presenting key outcomes of the FNFNES, for the first time, draw a remarkable picture of the modern diets of First Nations, along with the suite of environmental factors that are present in food and water in and around communities. One of the most remarkable results of the study—in addition to some dramatic new knowledge it developed particularly in regard to the extent of food insecurity among First Nations—is that it was sustained as a lively, vibrant partnership for more than a decade and is in fact continuing further with new ideas; for example, the new multidisciplinary study titled “Food, Environment, Health and Nutrition of First Nations Children and Youth” (http://www.fehncy.ca/).

In my role as an Indigenous physician assisting Indigenous peoples, I was in part hearing about their everyday concerns about the world around them. At the same time though, we were defining a new area of knowledge: “Indigenous environmental public health.” Indigenous peoples were feeling “a death by a thousand cuts” as little by little everything they knew and loved was changing. And at the same time, they recognized that their lands and waters were protective—were the seat of language, culture, memory, and genealogy, and even literally the sites of their very origin stories and stories of creation. Now their animals and fish—even air and weather—were subtly changing—and their access to and relationships with them were being challenged as well. The land is not as they remember it. Can they eat their country foods and ever feel the same as they did not long ago?

My work with Indigenous people often involves reassuring them that change is usually subtle—though occasionally catastrophic—and we *can* reconcile those changes. And if we cannot accept, we can endeavour to make change. Indigenous people are good at seeing that everything is connected. Environmental degradation affects so many different aspects of their lives—from their sense of security and connection and justice, to their everyday travelling, to their spiritual and emotional lives. They understand that they are in the exact same place as their ancestors, but in a different time.

Part of my work is to support their self-determination. If they’re concerned about cancer incidence in their village, I am too. If they don’t want their environment altered in order to make way for “progress,” I am supportive. In a way, Indigenous opinions—and their sovereignty—are increasingly butting up against Canadian realities. But can a scientist and an Elder sit comfortably together? Have a relationship? Decide on and share action and work? Even be good neighbours and friends and collaborators? I think that particularly now, with the example of the First Nations Food, Nutrition and Environment Study, this can happen in Canada. I think we can set the table and respectfully set opinions onto each other and do this over and over again. In fact, Canada is already a multicultural, pluralistic success story—we can add Indigenous realities into the mix. Canada has Indigenous roots. Water values, neurological manifestations, and stories of what the land used to be can sit side by side. The heart of treaties between settlers and Canada’s original peoples is an agreement to co-exist and walk together—but separate. Let us walk together for a while. I hope that you will find this issue of the *Canadian Journal of Public Health* informative, enjoyable, and instructive so that when you set up your joint research initiatives with Indigenous peoples, you do it with respect and with equity and treat it as an *ethical space* of continuing dialogue and growing new knowledge.

## Footnotes

1 “The ethical space, or the place of convergence of two societies with two worldviews, can also represent a location from which a meaningful dialogue can take place between communities towards the negotiation of a new research order that ethically engages different knowledge systems. These are knowledge systems embedded in communities under different political, historical, social, and economic realities.” (Ermine, 2000, p. 122)
